# The relationship of cannabis decriminalization in Colorado and cannabis use in individuals with alcohol use disorders

**DOI:** 10.1186/s42238-020-00018-0

**Published:** 2020-03-02

**Authors:** Jeremy T. Hua, Majid Afshar, Brendan J. Clark, Elizabeth J. Kovacs, Ellen L. Burnham

**Affiliations:** 1 Department of Medicine, University of Colorado School of Medicine, Aurora, CO USA; 2 Department of Medicine, Division of Pulmonary and Critical Care, Loyola University Chicago Health Sciences Campus, Chicago, IL USA; 3 Department of Medicine, Division of Pulmonary Sciences and Critical Care Medicine, University of Colorado School of Medicine, 12700 E. 19th St. C272, Aurora, CO 80045 USA; 4 Department of Surgery, Division of GI, Trauma, and Endocrine Surgery, University of Colorado School of Medicine, Aurora, CO USA

**Keywords:** Legislation, Cannabis, Tobacco, Alcoholism, Codependency, Dual use

## Abstract

**Objective:**

Over the past decade, cannabis use has become increasingly popular in states that include Colorado. During this time, alcohol use disorders (AUDs) and alcohol-related medical conditions have also been consistently recognized as public health problems with increasing prevalence in the state. Despite the widespread use of cannabis in Colorado, the epidemiology of cannabis use among those with AUDs has been poorly described. Therefore, we sought to examine cannabis use among individuals with likely AUDs and individuals with low-risk alcohol use during a time of major Colorado legislative changes before and after legalization of recreational cannabis in 2012.

**Methods:**

This study was a secondary data analysis conducted with information from 303 participants (80% male) in the Denver, CO metropolitan enrolled between August 2007 and April 2016 for studies related to alcohol and lung health. Of these participants, 188 (62%) were completing inpatient alcohol detoxification with likely AUDs. All participants completed the Alcohol Use Disorder Identification Test (AUDIT) to establish their likelihood of an AUD, and all had information on current cannabis use assessed by questionnaire and urine toxicology testing.

**Results:**

Individuals with likely AUDs more commonly used cannabis compared to control participants (42% vs 27%, *p* = 0.007). In multiple logistic regression analyses, participant type (likely AUD versus control), tobacco smoking, and age were significantly associated with cannabis smoking; however, the year of participant enrollment was not. Adjusted odds for cannabis use among participants with likely AUDs were 2.97 (1.51–5.82), *p* = 0.002, while odds for cannabis use among tobacco smokers were 3.67 (1.94–6.93), *p* < 0.0001. Among control participants, tobacco smoking increased odds of cannabis use seven-fold.

**Conclusions:**

Our findings highlight the exceptionally high odds of cannabis use among individuals with likely AUDs undergoing alcohol detoxification at a Colorado treatment facility before and after legalization of recreational cannabis. Targeted investigations into the medical and psychiatric consequences of combined alcohol and cannabis use are urgently needed to define its health impact in these vulnerable individuals.

## Introduction

As cannabis legislation has rapidly evolved in the United States, cannabis’ acceptability and use have increased steadily. The impact of widespread cannabis availability on its use is particularly important to delineate in individuals with alcohol use disorders (AUDs), who are at-risk for serious mental and medical health conditions, particularly liver disease and pulmonary infections (Caputo et al. 2012; Clark et al. 2013; Fernandez-Sola et al. 1995; Moss and Burnham 2006; Saitz et al. 1997). Although some studies indicating deleterious health outcomes from cannabis use have emerged, the evidence remains sparse (Lee and Hancox 2011; Committee on the Health Effects of Marijuana 2017; Pletcher et al. 2012). Both alcohol (Mayfield et al. 2013; Moss and Burnham 2006) and cannabinoids (Cabral et al. 2015) possess potent immunomodulatory effects, but their combined effects on health are not well understood. Epidemiologic investigations suggest an association between cannabis use and decreased severity of alcohol-related end-organ damage in such diseases as pancreatitis (Goyal et al. 2017) and liver disease (Adejumo et al. 2018). Precise mechanisms underlying protective effects of cannabis in alcohol-related diseases are not established, but the potential ability of cannabis to attenuate pro-inflammatory cytokine production necessary for progression of alcohol-related diseases has been postulated to play a role (Karoly et al. 2018). However, combined use of alcohol and cannabis may negatively impact public health, as dual use has been linked to the development of alcohol dependence (Midanik et al. 2007), increased health care utilization (John and Wu 2017), and an impaired ability to perform motor tasks such as driving (Downey et al. 2013). Compared to other states, Colorado ranks highly in both its use of alcohol and cannabis consumption. Data collected during 2013–2014 revealed that over 60% of adults reported alcohol consumption within the past month, and 6.9% were diagnosed with alcohol abuse or dependence (SAMHSA 2014) based on Diagnostic and Statistical Manual of Mental Disorders (DSM)-IV definitions. During this same period, cannabis use was reported in 16.80% of Colorado adults older than 26 years in the prior year, an increase from 10.73% in 2003 (SAMHSA 2014). Whether cannabis use in Colorado serves as a substitute or complement to alcohol use is unclear; results from published investigations involving US populations are conflicted (Subbaraman 2016), and may be related to characteristics of the study populations. For example, in recently sober patients with AUDs, continued cannabis use was associated with fewer days of alcohol abstinence (Subbaraman et al. 2017), while use of cannabis for medical indications has been linked to less problem drinking (Subbaraman and Kerr 2018).

Medical cannabis has been available for purchase in Colorado since 2000 (Colorado Constitution 2000) and recreational cannabis was decriminalized in late 2012 (Colorado Constitution 2012). Commercial cannabis sales were permitted beginning January 2014. As cannabis legalization has proceeded, delineating cannabis use patterns among individuals with substance abuse can identify opportunities for interventions to preserve health. Individuals who are already suffering psychiatric and medical consequences from excessive alcohol use represent a high-risk group that may perhaps be more vulnerable to the negative consequences of cannabis use. The alcohol research program at University of Colorado has been collecting data since 2007 from participants with AUDs, presenting an opportunity for a natural experiment designed to examine cannabis policy changes on cannabis use in this high-risk group. More specifically, the prevalence of cannabis use among those with likely AUDs in Colorado could be explored, as well as any effect modification related to tobacco use common in AUDs and in the setting of cannabis use (Agrawal et al. 2012; Falk et al. 2006). Further, the impact of legislative changes during this time period on the odds for cannabis use among Denver, Colorado adults with likely AUDs could be compared to a group of individuals without AUDs in the same geographic area. We hypothesized that cannabis use would become more prevalent in Denver after decriminalization, and that the increase in use would be greater among adults with likely AUDs.

## Methods

### Participants

Data were collected between August 2007 and April 2016 for on-going projects to examine the impact of AUDs on lung health (Burnham et al. 2012; Burnham et al. 2016; Burnham et al. 2011). Sources of data included surveys and laboratory test results collected from a cross section of Denver residents completing alcohol detoxification, and representative community control participants. Recruitment for the projects resulted in a total enrollment of 188 participants with likely AUDs and 115 healthy comparison participants. Enrollment of participants was driven by requirements of the parent studies’ design, including the need to coordinate inpatient visits to complete study-related screening and protocol elements detailed below. All participants enrolled during the 2007–16 period were included in this study. The Colorado Multiple Institutional Review Board approved the study. All participants provided written informed consent to have their data used in future research studies. Participants with likely AUDs were recruited from an alcohol detoxification facility, Denver Comprehensive Addictions Rehabilitation and Evaluation Services (CARES), affiliated with Denver Health and Hospital Administration in Denver, CO. We focused on identifying participants without evidence of other nonmedical or illicit substance use. Trained research coordinators visited the facility to screen sober participants who had recently completed alcohol detoxification, and who had one or more prior admissions for alcohol detoxification. Unhealthy alcohol use was assessed with the Alcohol Use Disorders Identification Test (AUDIT) (Babor et al. 2006). Participants with AUDIT scores of ≥8 in men or ≥ 5 in women who had used alcohol within the prior 7 days and who were ≥ 21 years old were included. Participants reporting a prior serious medical history requiring chronic medications, pregnancy, or substance use other than alcohol, cannabis, or tobacco were excluded (Burnham et al. 2012; Burnham et al. 2016; Burnham et al. 2011). Research participation did not interfere with usual care at the detoxification facility. The comparison group with low-risk alcohol use was identified using a combination of internet and print advertisements in the health system’s catchment area. Participants with AUDIT scores of < 8 in men or < 5 in women, and who were ≥ 21 years old were included. Exclusion criteria were identical to those for alcohol detoxification eligibility.

### Protocol

Research protocols were conducted in the University of Colorado Hospital’s Clinical and Translational Research Center (UCH-CTRC, Aurora, CO). All participants completed questionnaires regarding alcohol, tobacco, and drug use at the time of enrollment. Subsequently, a clinical evaluation including a history and physical exam, baseline laboratory testing, a chest radiograph, spirometry, and urine toxicology screen was performed. Criteria excluding participants from enrollment after the clinical evaluation were as follows (Burnham et al. 2012; Burnham et al. 2016; Burnham et al. 2011): a prior medical history of liver disease or cirrhosis, total bilirubin > 2.0 mg/dL, or albumin < 3 g/dL; prior medical history of myocardial infarction or congestive heart failure; prior medical history of end-stage renal disease or serum creatinine > 3 mg/dL; positive urine toxicology screen for opiates, cocaine, or methamphetamines; history of diabetes mellitus; history of chronic obstructive pulmonary disease (COPD) or asthma; history of HIV; peripheral white blood cell count of less than 3000/μL; abnormal chest radiograph; spirometry of < 60% predicted for either FEV_1_ and FVC; use of systemic antibiotics for any reason in the 4 weeks; or current pregnancy. Nineteen likely AUD participants were excluded (eight for positive toxicology screens, four for abnormal chest radiographs, and seven for other reasons) after intake assessment due to stipulations in the parent studies’ protocols. One comparison participant changed his mind about participating in the study, and was withdrawn.

Given the prevalence of tobacco use among individuals with AUDs (Batel et al. 1995; DiFranza and Guerrera 1990; Falk et al. 2006; Zacny 1990), as well as the common combined use of tobacco and cannabis (Agrawal et al. 2012; Conway et al. 2017), additional detailed information regarding the use of smoked products was ascertained. To characterize the use of tobacco and/or cannabis, participants were asked whether they actively smoked tobacco or used cannabis in any formulation. If yes, the pack-year use of tobacco and total number of years engaged in cannabis use was recorded. Individuals also provided a urine sample to qualitatively test for cannabinoids. Urine testing was performed with the Alere iCassette DX Drug Test kit (ACON Laboratories, Inc., San Diego, CA), a chromatographic immunoassay for the qualitative detection of multiple drugs and metabolites in urine. For cannabis, the calibrator was Δ-9-THC-COOH at a cutoff of 50 ng/mL. Cannabis use in this study was defined either as reported current use, or if the urine toxicology screen was positive for cannabinoids, given that less regular cannabis consumers may not have tested positive (Desrosiers et al. 2014). In addition to recording the participants’ year of enrollment during the time of data collection, participants were subdivided into three time intervals based on when data collection began, and when significant legislative changes in Colorado related to cannabis occurred: (1) August 2007 to October 2012, prior to cannabis legalization for recreational use, (2) November 2012 to December 2013, after legalization for recreational use but before retail sales were legal, and (3) January 2014 to April 2016, after legalization for sales by retail businesses.

### Statistical analyses

Initial comparisons of baseline demographic information and cannabis use history between likely AUD participants and comparison participants were performed using Chi-square tests, or Fisher’s exact test for proportions. The primary outcome was the odds ratio (OR) for cannabis use. The following variables were examined in multivariable logistic regression models: likely AUD status (yes or no), smoking status (current or not current), age (per year), sex, race (Caucasian versus other), and ethnicity (Hispanic versus non-Hispanic). The timing of participant enrollment was included in the models in one of two ways: (1) participants were classified by their enrollment year as belonging in one of the three time periods corresponding to Colorado legislative changes (i.e. 2007–11, 2012–13, or 2014–16), or (2) year of participant enrollment was considered a continuous variable. Interaction terms, including participant type*tobacco smoking, and participant type*time of enrollment were also examined in the models.

The relationship of between the timing of participants’ enrollment and AUDIT scores was also explored using linear regression analyses. Since AUDIT scores were not normally distributed across the entire cohort, separate models were created for likely AUD and control participants. Included in the models for both participant types were smoking status, age, sex, race, and ethnicity. The time period or year of participant enrollment was also included in the models in one of the two ways outlined above.

Statistical analyses were performed using JMP® Statistical Software for Windows version 12.0 (SAS Institute Inc., Cary, NC).

## Results

### Study population

During the study period, 303 participants were enrolled, including 188 participants with likely AUDs who had recently completed alcohol detoxification, along with 115 comparison participants. All comparison participants were classified as low-risk alcohol consumers based on their AUDIT scores. Participants with likely AUDs were older and predominantly male (Table 1). Fewer were Caucasian race, and more were likely to be of Hispanic ethnicity. The health of participants, assessed during screening, did not differ significantly between enrolled participants with likely AUDs and the comparison group including serum total white blood cell count, creatinine, and total bilirubin. Further, baseline spirometry obtained to assess lung physiology was also not different between groups. Tobacco use was more common among the likely AUD group, but pack-year history between groups was similar. The average AUDIT score for the likely AUD group was 28 (95% CI [26.7, 29]), and 81% (*n* = 153) had scores > 20, placing them at the highest risk for AUDs.

**Table 1:** Description of the Study Population

	By Group		*P*
	Comparison	Likely AUD	
	*N* = 115	*N* = 188	
Age (years)	40.6 ± 8.0	43.4 ± 6.8	*0.002*
Number of men (%)	79 (69%)	164 (87%)	*0.0003*
-- Race/Ethnicity, n (%) --			
Caucasian	87 (76%)	118 (62%)	*0.01*
African-American	22 (19%)	26 (14%)	*0.20*
Hispanic/Latino	18 (16%))	62 (33%)	*0.001*
Asian	4 (4%)	1 (0.6%)	*0.05*
Pacific Islander	1 (1%)	2 (1%)	*0.88*
American Indian	3 (3%)	42 (22%)	*< 0.0001*
AUDIT Score	2.3 ± 1.8	27.9 ± 8.1	*< 0.0001*
Report Tobacco Use, n (%)	62 (54%)	127 (67%)	*0.03*
Tobacco History (Pack-Years)^a^	18.2 ± 12.8	15.2 ± 13.9	*0.16*
Positive Cannabis Use, n (%)^b^	31 (27%)	80 (42%)	*0.007*
Cannabis History (Years)^a^	10.3 ± 10.1	11.6 ± 10.2	*0.50*

Values reported in mean ± standard deviation

*Abbreviations*: *AUDIT* Alcohol Use Disorder Identification Test

^a^Average for subjects reporting tobacco/cannabis use

^b^Positive cannabis use defined as either by self-report or by positive urine toxicology screen

### Relationship of cannabis use with alcohol detoxification

Concordance in reported cannabis use versus urine toxicology testing was observed in 72% (72/99) of all participants, suggesting heavy cannabis consumption in the majority of cannabis users (Desrosiers et al. 2014). The case-rate of cannabis use differed between the likely AUD group compared to the comparison group (42% vs 27%, Table 1). Duration of cannabis consumption between the likely AUD group and comparison group did not differ, and averaged just over a decade.

### Cannabis use over ten years among participants

**Fig. 1:**
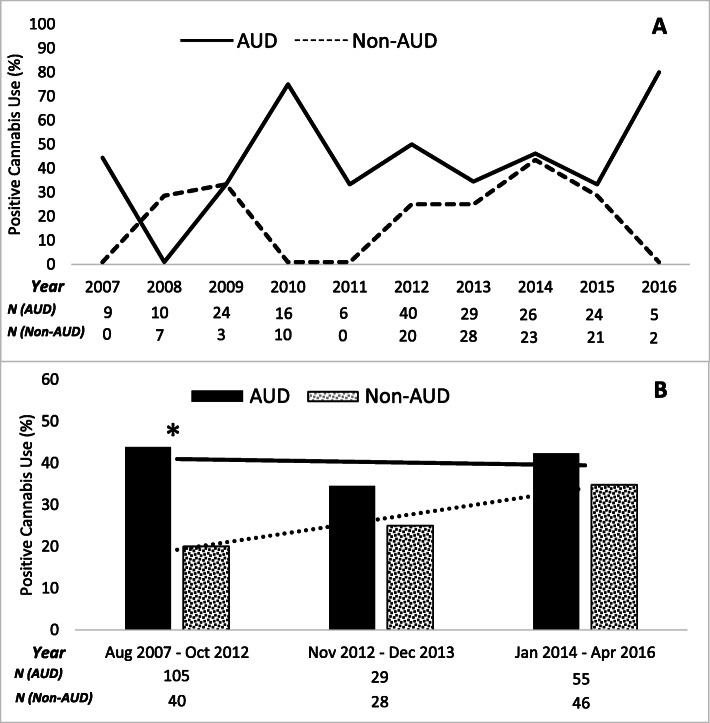
Cannabis use in Denver, Colorado among participants with likely alcohol use disorders (AUDs) compared to participants with low risk alcohol use (Non-AUD) during **a** each year of study enrollment from 2007 to 2016; and **b** three time-intervals of study enrollment corresponding to pertinent legislative change: prior to cannabis legalization for recreational use (August 2007 to October 2012), after legalization for recreational use (November 2012 to December 2013), and after legalization for sales by retail businesses (January 2014 to April 2016). *Denotes significant group differences in cannabis use (*p* = 0.006 by Chi-square analysis) for the specified time-interval

Over the duration of the study period, cannabis use was more prevalent among participants with likely AUDs nearly every year (Fig. 1a). Moreover, there was a relative increase in the proportion of cannabis use among non-AUD comparison participants after cannabis was legalized for personal possession and retail sales. In comparing cannabis use among the group with likely AUDs to the non-AUD comparison group within each of the three time periods in unadjusted analyses (Fig. 1b), cannabis use during the 2007–2012 period was greater in the likely AUD group, but the proportion of cannabis users was similar between groups during the two subsequent time periods.

Multivariable logistic regression analyses were performed to determine the association between participant variables, including timing of enrollment, and cannabis use. A significant interaction was noted between likely AUD and tobacco use (interaction term, *p* = 0.007). Therefore, the interaction term was included in the final multivariable models. We observed that participant group (likely AUD versus control), tobacco use, the interaction term of participant group*tobacco use, and age were each significantly associated with cannabis use, but not time period of enrollment (Table 2). Being a participant with likely AUD was associated with odds for cannabis use of 2.97 (1.51–5.82), *p* = 0.002, adjusting for factors listed in the model. In contrast, comparison participant status was associated with odds for cannabis use of 0.34 (0.17–0.66), *p* = 0.002. Tobacco smoking was associated with odds for cannabis use of 3.67 (1.94–6.93), *p* < 0.0001. Among participants with AUDs, those who smoked cigarettes increased odds of cannabis use by 1.3, while among control participants, those who smoked cigarettes increased odds of cannabis use by 7.4. With each additional year of age, odds for cannabis use diminished by 5% (2–9%). Odds for cannabis use between time periods were not significantly different (odds for cannabis use between 2007 and 09 and 2012–13 were 1.18 (0.63–2.21); odds for cannabis use between 2007 and 09 and 2014–16 were 1.45 (0.76–2.80)). In separate models, interaction terms including participant type and time period of enrollment were not significant (Supplemental Table I). If instead of time period of enrollment, year of enrollment was included in the model as a continuous variable, it did not appreciably change fit of the model and was not significantly associated with cannabis use (*p* = 0.28, Supplemental Table II).

**Table 2:** Multiple logistic regression analysis to determine predictors of cannabis use among the entire cohort (*n* = 303)

Term in Model	Estimate	Standard Error	Chi Square	*P* value
Intercept	0.73119581	0.8310458	0.77	0.3789
Participant Group, likely AUD vs Control	0.5439573	0.1718429	10.02	0.0015
Enrollment 2012–2013 vs 2007-11^a^	0.16683894	0.3200676	0.27	0.6022
Enrollment 2014–2016 vs 2007-11^a^	0.37500419	0.3341555	1.26	0.2618
Sex, Women vs Men	− 0.2250948	0.1772926	1.61	0.2042
Age in years	− 0.047305	0.0186226	6.45	0.0111
Tobacco Use, no vs yes	−0.6498842	0.1625191	15.99	<.0001
Hispanic/Latino, no vs yes	0.1710905	0.1533545	1.24	0.2646
White, no vs yes	0.06257438	0.1368995	0.21	0.6476
Subject Group*Tobacco Use (interaction)	0.435371	0.1642911	7.02	0.0080

^a^The three time-intervals of study enrollment correspond to pertinent legislative change: prior to cannabis legalization for recreational use (August 2007 to October 2012), after legalization for recreational use (November 2012 to December 2013), and after legalization for sales by retail businesses (January 2014 to April 2016)

### AUDIT scores over ten years among participants

Given the potential for cannabis legislation to have influenced alcohol consumption habits, or for changes in Colorado’s population to have occurred over the years participants were being enrolled, linear regression models to examine the relationship between time period of enrollment on AUDIT scores were created. Given that AUDIT scores across the entire study population were not normally distributed, separate models for the likely AUD participants and the comparison group were created. Table 3 highlights the parameter estimates for each term in predicting AUDIT scores. Time period of enrollment was not significantly associated with AUDIT scores for either likely AUD participants or controls, adjusting for age, sex, tobacco use, or race/ethnicity. Results were similar in separate models where year of enrollment was coded as a continuous variable (Supplemental Tables III and IV).

**Table 3:** Multivariable linear regression analyses to determine relationship between clinical variables and AUDIT scores in likely AUD participants and controls

Term in Model	Estimate	Standard Error	t Ratio	Prob > |t|
**Relationship between Clinical Variables and AUDIT Among Likely AUD Participants, n = 188**				
Intercept	33.576527	4.13464	8.12	<.0001
Enrollment 2012–13 vs 2007-11^a^	1.3564002	1.422927	0.95	0.3417
Enrollment 2014–16 vs 2007-11^a^	0.9415975	1.546689	0.61	0.5434
Age in years	−0.151301	0.090466	−1.67	0.0962
Sex, women vs men	−0.491879	0.90464	−0.54	0.5873
Tobacco Use, no vs yes	0.3890036	0.656083	0.59	0.5540
White, no vs yes	−0.046453	0.635463	−0.07	0.9418
Hispanic/Latino, no vs yes	−0.388884	0.673469	−0.58	0.5644
**Relationship between Clinical Variables and AUDIT Among Control Participants, n = 115**				
Intercept	2.5989497	1.011763	2.57	0.0116
Enrollment 2012–13 vs 2007-11^a^	0.5679863	0.49472	1.15	0.2535
Enrollment 2014–2016 vs 2007-11^a^	0.4031983	0.502478	0.80	0.4241
Age in years	−0.024706	0.022206	−1.11	0.2684
Sex, women vs men	−0.080338	0.19504	−0.41	0.6812
Tobacco Use, no vs yes	−0.040731	0.184397	−0.22	0.8256
White, no vs yes	−0.112426	0.214492	−0.52	0.6013
Hispanic/Latino, no vs yes	0.3227031	0.247132	1.31	0.1945

^a^ The three time-intervals of study enrollment correspond to pertinent legislative change: prior to cannabis legalization for recreational use (August 2007 to October 2012), after legalization for recreational use (November 2012 to December 2013), and after legalization for sales by retail businesses (January 2014 to April 2016)

## Discussion

In this study, we determined that over 40% of participants undergoing alcohol detoxification with likely AUDs, who were otherwise healthy based on detailed health screening and not using illicit drugs, consume cannabis in the Denver, CO metropolitan area. In contrast to our initial hypothesis, the odds for cannabis consumption among individuals with likely AUDs has been remarkably high, but was not significantly associated with the year of enrollment, although major legislative changes were occurring between 2007 and 2016. Our investigations provide longitudinal data regarding cannabis use over a ten-year period in a well-characterized urban cohort with likely AUDs in a state that where cannabis laws have undergone rapid evolution. To our knowledge, similar studies examining cannabis use trends in populations with likely alcohol dependence have not been conducted in other states promoting early cannabis legalization. Importantly, individuals we enrolled with likely AUDs often smoked cigarettes but were not using other illicit substances routinely. Our observations suggest that individuals primarily seeking alcohol (but not other substance) detoxification have a high odds for cannabis consumption, deserve further scrutiny regarding the psychological and medical effects of their dual use. Further, tobacco smoking was also associated with substantially increased odds for cannabis consumption among both participant types. Our findings may be particularly relevant in parts of the United States where cannabis laws are evolving. Importantly, it is reasonable to expect that sequelae of co-dependence will be magnified with the further commercialization of the cannabis industry, as healthcare utilization for both cannabis- and alcohol-related medical conditions remains a considerable concern (Chavez et al. 2016; John and Wu 2017; Mokdad et al. 2018).

Since both alcohol and cannabis have reported immunomodulatory effects, it is critical to consider their combined effects on health. Moreover, as current tobacco use was more likely to be reported by cannabis-using participants, this further underscores the possibility of alcohol-cannabis-tobacco users as a cohort at risk for lung health problems in particular (Bailey et al. 2019; Gaydos et al. 2016). Alcohol’s immune effects are related to exposure habits. Chronic alcohol consumption (as in our population with likely AUDs) has been associated with a promotion of inflammation and impaired anti-inflammatory mediator activity (reviewed in (Szabo and Saha 2015)). On the other hand, cannabis use is believed to suppress inflammation, characterized by induction of apoptosis in activated immune cells, suppression of cytokines and chemokines at sites of inflammation, and upregulation of adaptive immunity (Cabral et al. 2015; Nagarkatti et al. 2009; Tashkin et al. 2002). Although it is likely that dual alcohol and cannabis use can affect all organs, potential deleterious effects related to psychiatric and behavioral health are the best studied. Dual use of alcohol and cannabis has been associated with AUD development, characterized by increased severity, and longer duration (Midanik et al. 2007). Dual dependency on cannabis and alcohol has been associated with increased healthcare utilization (John and Wu 2017). Controlled studies to measure the effects of combined alcohol and cannabis consumption on motor tasks (e.g. driving simulations) indicate that dual use objectively and additively impairs driving performance (Hartman et al. 2015; Ramaekers et al. 2011). Further, combined consumption of alcohol and cannabis in controlled settings results in higher blood levels of cannabinoids than cannabis consumed without alcohol (Downey et al. 2013; Lukas and Orozco 2001) that may potentiate the acute neurocognitive effects of cannabis on the consumer. Despite these reports, however, the potential for cannabis to “counterbalance” immune effects of harmful alcohol consumption should also be considered. Cross-sectional investigations in gastrointestinal diseases including alcohol-related pancreatitis (Goyal et al. 2017) and liver disease (Adejumo et al. 2018) argue that immune-modulating effects of combined cannabis and alcohol can perhaps attenuate severity of illness and preserve organ function, though mechanisms remain ill-defined. Downstream effects of cannabis on metabolism, perhaps mediated through its immune effects, are highlighted in epidemiologic reports of a diminished prevalence of obesity with lower body mass index in US adults who use cannabis (Le Strat and Le Foll 2011). Separate investigations have further noted a lower age-adjusted prevalence of diabetes and lower serum C-reactive protein (Rajavashisth et al. 2012) and reduced prevalence of non-alcoholic fatty liver disease (Adejumo et al. 2017) among cannabis users compared to non-users. Given the prevalence of dual alcohol and cannabis use, delineating its purported risks and benefits on health should be a priority.

Coloradans have reported increased cannabis use over time (Schuermeyer et al. 2014). In individuals with likely AUDs, our observations were comparable to prior studies reporting a 26–48% point prevalence of cannabis use (Anderson et al. 2018; Fuster et al. 2017; Mojarrad et al. 2014). However, our data did not suggest that cannabis use is increasing over time among people undergoing alcohol detoxification in the state (Guttmannova et al. 2016). Notably, the point prevalence of approximately 40% in the likely AUD cohort falls on the high end of previous reports (Anderson et al. 2018; Fuster et al. 2017; Mojarrad et al. 2014). In these earlier investigations involving AUD participants, many endorsed other illicit substance use or dependence along with cannabis use. However, in our likely AUD cohort, history and urine toxicology screens indicated that illicit use was either absent, or too infrequent to detect. Moreover, compared to the US locations where these studies were conducted (Massachusetts (Mojarrad et al. 2014) and Ohio (Anderson et al. 2018)), medical cannabis has been available in Colorado substantially longer, perhaps explaining the relatively high point prevalence of cannabis use we observed. One other possibility for the already-high prevalence of cannabis use prior to major legislative changes we observed among individuals with AUDs is that these individuals are not influenced by the risk or stigma associated with cannabis use.

Overall, the rate of cannabis use in our study’s healthy comparison group (27%) over the entire study period was higher than published rates (16% prior-year use) in a 2014 Colorado-wide survey of adults over 26 years (SAMHSA 2014), potentially reflecting the healthy volunteer effect in clinical studies. Interestingly, cannabis users in the comparison group had consumed cannabis for a similar length of time as the cohort with likely AUDs. As mentioned above, this finding could be related to legalization of medical cannabis in 2000 that facilitated acquisition of the drug for all Colorado adults, regardless of AUD or other drug use (1) (Colorado Constitution 2000). Observing that average cannabis use duration was a decade or more in both participant types suggests that residents of the state have incorporated cannabis use into their everyday lives as its perceived risk has diminished (Schuermeyer et al. 2014). Certainly, the trend we observed for relatively increased use among the comparison group may not be generalizable to the broader Colorado population given our small sample size, and could perhaps be related to an increase in numbers of cannabis users moving to Colorado from other states (Kim et al. 2016). However, in analyses examining the relationship between time period of enrollment on AUDIT scores, adjusting for other cohort characteristics including age, sex, and tobacco use we found that AUDIT scores did not change significantly over the study period in either group. The relative stability in AUDIT scores over time makes it less likely that factors such as a major changes in characteristics of the enrolled population (such as with migration) influenced our results. To further explore the possibility that legislative changes have played a role in increased cannabis consumption, two other data sources can be examined. Data from the Behavioral Risk Factor Surveillance System (BRFSS), a federally-funded random telephone survey of Colorado residents ≥18 years of age, demonstrated an increase in daily or near-daily cannabis use between 2016 and 2017 of 6.4 to 7.6% (*p* = 0.02) (BRFSS 2019). In parallel, the National Survey on Drug Use and Health (NSDUH) reported that 15.9% of the population consumed marijuana in 2018, a value higher than percentages from 2002 to 2017 (Lipari and Park-Lee 2018). Importantly, BRFSS and NSDUH data sources further highlighted the inverse relationship between adult age and cannabis consumption that we confirmed in our analyses. Among adults aged 26–34, most closely aligned to the healthy comparison group in this study, cannabis consumption prevalence in the BRFSS was 19.4% in 2016 and rose to 26.4% in 2017. Therefore, the point prevalence of cannabis use, in our comparison cohort are in keeping with additional larger data sources collected from Colorado residents.

Previous investigations suggest that the relationship between cannabis and alcohol consumption is complex. As mentioned, parallel studies including individuals with harmful alcohol use have not been conducted at the state level. However, studies exploring outcomes among dual alcohol and cannabis users in other populations do exist. One systematic review (Guttmannova et al. 2016) evaluated fifteen investigations to assess the impact of changing cannabis legislation on alcohol use, and included studies with varied populations including (for example) national samples, teenagers, and cannabis dispensary customers. Experts authoring this review recommended additional efforts to compare outcomes between states that have enacted pro-cannabis legislation to those who have not, emphasizing the importance of additional single state studies (using, for example, repeated cross-sectional samples) to assess the impact of evolving cannabis policies on deviations in substance use outcomes over time. Moreover, these authors emphasized that cannabis policies were likely to influence alcohol use in complex ways that will be challenging to study. One important area surrounding dual alcohol and cannabis use deserving of further clarification centers around the question of whether cannabis acts as a substitute, or a complement, to alcohol use. One review of 39 available studies found data to be mixed in regards to habits of cannabis use in conjunction with alcohol (Subbaraman 2016). However, closer examination of unique populations of dual users in context is illuminating. In one cohort of recently sober individuals with AUDs followed in the context of a longitudinal, randomized clinical trial, cannabis use was associated with reduced abstinence at the end of a 68 week intervention period (Subbaraman et al. 2017). In a separate cohort of community-dwelling individuals from the State of Washington who endorsed use of both alcohol and cannabis in the past 12 months, cannabis use for a presumed medical indication was associated with lower AUDIT and AUDIT problem scores, compared to cannabis users without a medical recommendation (Subbaraman and Kerr 2018). Since AUDIT data from our cohort with likely AUDs was not appreciably related to the year of participant enrollment, we presently have no reason to believe that our participants were substituting cannabis for alcohol consumption. Our study and others indicate that reasons for dual alcohol and cannabis user are contextual, and perhaps related to the neurobiology of the consumer.

Our work is not without limitations. First, there were baseline sex differences between groups, in part driven by the smaller women’s facility at Denver CARES. Moreover, data from our study are insufficient to understand the impact of race and ethnicity on cannabis use, though the AUDs in nearly all racial and ethnic subgroups has risen in the last decade (Grant et al. 2017). Nevertheless, these limitations are tempered by the data being acquired from a population of well-characterized individuals who are extremely likely to experience health harm from alcohol use alone, and in whom use of cannabis may represent excessive health risk. In this cohort, it was remarkable that cannabis use and alcohol abuse, without the abuse of other substances, occurred so commonly. Year of enrollment, corresponding to a time period of changing cannabis legalization was not associated with major changes in cannabis consumption among those with likely AUDs. Nevertheless, these participants’ overall health may have been affected. Longitudinal examinations were not performed in the cohort with likely AUDs, however, precluding an assessment of how continued harmful alcohol use and cannabis use influenced overall health. These will be important to consider in the future. Secondly, we did not assess the quantity or route(s) of cannabis consumption, or reasons for cannabis consumption (e.g. medical versus recreational) as these data were initially collected for parent studies focused on lung health, prior to fully appreciating how cannabis legislation would evolve. Participants self-reporting cannabis use but who had negative urine toxicology tests could have been occasional, rather than frequent, cannabis smokers, potentially explaining the discordance we observed between cannabis assessments (Desrosiers et al. 2014). Whether the percentage of individuals who were using cannabis purchased legally (i.e. with a medical prescription) changed over time with changes in the law is not known, but based on cost considerations alone, it seems less likely that medical users would switch to purchasing recreational products given the higher taxes associated with the latter (Van Dyke et al. 2018). Future investigations can benefit from a more detailed inquiry into frequency and quantity of cannabis use (Grotenhermen 2003; Monte et al. 2015). Since health effects related to cannabis may logically be related to problematic cannabis use, studies should employ DSM-5 criteria to define whether cannabis use disorders are becoming more prevalent over time, and whether they are associated with AUDs. Moreover, we acknowledge that low-risk drinkers in the study were identified by AUDIT only, and a clinical interview may have provided more definite information with regard to alcohol consumption habits. However, low-risk drinkers must have had a normal comprehensive metabolic panel and medical screening prior to their inclusion, making active harmful alcohol use among this group less likely. Finally, we acknowledge that the single-center design may not be representative of alcohol detoxification centers elsewhere in the US. This feature is tempered by our ability to collect data for a lengthy time period from a stable, urban population.

As cannabis use increases nationwide and concurrent rates of unhealthy alcohol consumption are climbing, particularly in Colorado and other states, results of this study support the public health efforts aimed at the prevention and treatment of substance use disorders and their medical sequelae. Additionally, future studies that address the effect modification from cannabis use on unhealthy alcohol consumption have the potential to substantially impact health outcomes.

## Supplementary information

**Additional file 1: Supplemental Table I.** Multiple logistic regression analysis to determine predictors of cannabis use among the entire cohort (*n* = 303), including interaction terms between participant type and time period of enrollment. **Supplemental Table II.** Multiple logistic regression analysis to determine predictors of cannabis use among the entire cohort (*n* = 303), using time of enrollment as a continuous variable. **Supplemental Table III.** Linear regression analysis to determine the association between likely AUD participants’ characteristics and AUDIT scores. **Supplemental Table IV.** Linear regression analysis to determine the association between control participants’ characteristics and AUDIT scores.

## Data Availability

The datasets used and/or analyzed during the current study are available from the corresponding author on reasonable request.

## Abbreviations

AUDIT: Alcohol Use Disorders Identification Test
AUDs: Alcohol use disorders
Denver CARES: Denver Comprehensive Addictions Rehabilitation and Evaluation Services
DSM: Diagnostic and Statistical Manual of Mental Disorders
OR: Odds Ratio
THC: Tetrahydrocannabinol
UCH-CTRC: University of Colorado Hospital Clinical and Translational Research Center
