# Supplementary material for: The relationship of cannabis decriminalization in Colorado and cannabis use in individuals with alcohol use disorders
Source: J Cannabis Res. 2020 Mar 2;2:13. doi: 10.1186/s42238-020-00018-0 (PMC7819320; doi:10.1186/s42238-020-00018-0)
Supplement: Supplementary file 1 — Additional file 1: Supplemental Table I. Multiple logistic regression analysis to determine predictors of cannabis use among the entire cohort (n = 303), including interaction terms between participant type and time period of enrollment. Supplemental Table II. Multiple logistic regression analysis to determine predictors of cannabis use among the entire cohort (n = 303), using time of enrollment as a continuous variable. Supplemental Table III. Linear regression analysis to determine the association between likely AUD participants’ characteristics and AUDIT scores. Supplemental Table IV. Linear regression analysis to determine the association between control participants’ characteristics and AUDIT scores. [file 42238_2020_18_MOESM1_ESM.docx]

**The Relationship of Cannabis Decriminalization in Colorado and**

**Cannabis Use in Individuals with Alcohol Use Disorders**

Jeremy T. Hua, MD, Majid Afshar, MD, MS, Brendan J. Clark, MD, MS,

Elizabeth J. Kovacs, PhD, Ellen L. Burnham, MD, MS

**Supplemental Tables**

**Supplemental Table I.** Multiple logistic regression analysis to determine predictors of cannabis use among the entire cohort (n=303), including interaction terms between participant type and time period of enrollment.

| **Term in Model** | **Parameter Estimate** | **Standard Error** | **Chi Square** | **P value** |
| --- | --- | --- | --- | --- |
| Intercept | 0.61444579 | 0.866271 | 0.50 | 0.4781 |
| Participant Group  (likely AUD versus Control) | 0.59615281 | 0.1834193 | 10.56 | 0.0012* |
| Enrollment 2012-2013 v 2007-11^a^ | 0.33193408 | 0.4244245 | 0.61 | 0.4342 |
| Enrollment 2014-2016 v 2007-11^a^ | 0.6128639 | 0.4259593 | 2.07 | 0.1502 |
| Sex, women v men | -0.2173994 | 0.1783898 | 1.49 | 0.2230 |
| Age in years | -0.048976 | 0.0187392 | 6.83 | 0.0090* |
| Tobacco Use, no v yes | -0.6664557 | 0.1650608 | 16.30 | <.0001* |
| Hispanic/Latino, no v yes | 0.14730259 | 0.1544387 | 0.91 | 0.3402 |
| White, no v yes | 0.06569868 | 0.1372866 | 0.23 | 0.6323 |
| Participant Group*Tobacco Use (interaction) | 0.45414848 | 0.1670293 | 7.39 | 0.0065* |
| Participant group*  2012-2013 v 2007-11^a^ (interaction) | -0.1920383 | 0.4241722 | 0.20 | 0.6507 |
| Participant group*  2014-2016 v 2007-11^a^ (interaction) | -0.4878356 | 0.4275522 | 1.30 | 0.2539 |

^a^ The three time-intervals of study enrollment correspond to pertinent legislative change: prior to cannabis legalization for recreational use (August 2007 to October 2012), after legalization for recreational use (November 2012 to December 2013), and after legalization for sales by retail businesses (January 2014 to April 2016).

**Supplemental Table II.** Multiple logistic regression analysis to determine predictors of cannabis use among the entire cohort (n=303), using time of enrollment as a continuous variable.

| **Term in model** | **Parameter Estimate** | **Standard Error** | **Chi Square** | **P value** |
| --- | --- | --- | --- | --- |
| Intercept | -121.62455 | 113.34423 | 1.15 | 0.2832 |
| Participant Group  (likely AUD versus Control) | 0.54663754 | 0.1721558 | 10.08 | 0.0015* |
| Sex, women v men | -0.2209474 | 0.1772079 | 1.55 | 0.2125 |
| Age in years | -0.0475836 | 0.0186012 | 6.54 | 0.0105* |
| Tobacco Use, no v yes | -0.648879 | 0.1624749 | 15.95 | <.0001* |
| HIspanic/Latino, no v yes | 0.16865624 | 0.1530319 | 1.21 | 0.2704 |
| White, no v yes | 0.05925807 | 0.1367847 | 0.19 | 0.6649 |
| Participant Group*Tobacco Use (interaction) | 0.44018859 | 0.1641969 | 7.19 | 0.0073* |
| Year of participant enrollment | 0.06090838 | 0.056281 | 1.17 | 0.2792 |

**Supplemental Table III.** Linear regression analysis to determine the association between likely AUD participants’ characteristics and AUDIT scores.

| **Term** | **Parameter Estimate** | **Standard Error** | **t Ratio** | **Prob>\|t\|** |
| --- | --- | --- | --- | --- |
| Intercept | -408.0366 | 507.6256 | -0.80 | 0.4226 |
| Year participant enrolled | 0.2198018 | 0.252039 | 0.87 | 0.3843 |
| Sex, women v men | -0.478851 | 0.902752 | -0.53 | 0.5965 |
| Tobacco Use, no v yes | 0.4118619 | 0.653982 | 0.63 | 0.5296 |
| HIspanic/Latino, no v yes | -0.342679 | 0.669191 | -0.51 | 0.6092 |
| White, no v yes | -0.051265 | 0.634061 | -0.08 | 0.9356 |
| Age in years | -0.147455 | 0.089836 | -1.64 | 0.1024 |

**Supplemental Table IV.** Linear regression analysis to determine the association between control participants’ characteristics and AUDIT scores.

| **Term** | **Parameter Estimate** | **Standard Error** | **t Ratio** | **Prob>\|t\|** |
| --- | --- | --- | --- | --- |
| Intercept | -140.5888 | 179.0232 | -0.79 | 0.4340 |
| Year enrolled | 0.0713495 | 0.088926 | 0.80 | 0.4241 |
| Sex, women v men | -0.077688 | 0.196044 | -0.40 | 0.6927 |
| Tobacco Use, no v yes | -0.04592 | 0.183925 | -0.25 | 0.8033 |
| HIspanic/Latino, no v yes | 0.3128026 | 0.24677 | 1.27 | 0.2077 |
| White, no v yes | -0.094116 | 0.213938 | -0.44 | 0.6609 |
| Age in years | -0.024832 | 0.022184 | -1.12 | 0.2655 |
